# Long Waiting Times for Elective Hospital Care – Breaking the Vicious Circle by Abandoning Prioritisation

**DOI:** 10.15171/ijhpm.2019.84

**Published:** 2019-10-30

**Authors:** Solbjørg Makalani Myrtveit Sæther, Torhild Heggestad, John-Helge Heimdal, Magne Myrtveit

**Affiliations:** ^1^Department of Health Promotion, Norwegian Institute of Public Health, Bergen, Norway.; ^2^Department of Clinical Science, University of Bergen, Bergen, Norway.; ^3^Department of Research and Development, Haukeland University Hospital, Bergen, Norway.; ^4^Department of Clinical Medicine, University of Bergen, Bergen, Norway.; ^5^Clinic of Surgery, Haukeland University Hospital, Bergen, Norway.; ^6^Dynaplan AS, Manger, Norway (https://www.dynaplan.com/en/).

**Keywords:** Appointment Allocation, Waiting Time, Waiting List Management, Prioritisation, Dynaplan Smia

## Abstract

**Background:** Policies assigning low-priority patients treatment delays for care, in order to make room for patients of higher priority arriving later, are common in secondary healthcare services today. Alternatively, each new patient could be granted the first available appointment. We aimed to investigate whether prioritisation can be part of the reason why waiting times for care are often long, and to describe how departments can improve their waiting situation by changing away from prioritisation.

**Methods:** We used patient flow data from 2015 at the Department of Otorhinolaryngology, Haukeland University Hospital, Norway. In Dynaplan Smia, Dynaplan AS, dynamic simulations were used to compare how waiting time, size and shape of the waiting list, and capacity utilisation developed with and without prioritisation. Simulations were started from the actual waiting list at the beginning of 2015, and from an empty waiting list (simulating a new department with no initial patient backlog).

**Results:** From an empty waiting list and with capacity equal to demand, waiting times were built 7 times longer when prioritising than when not. Prioritisation also led to poor resource utilisation and short-lived effects of extra capacity. Departments where prioritisation is causing long waits can improve their situation by temporarily bringing capacity above demand and introducing "first come, first served" instead of prioritisation.

**Conclusion:** A poor appointment allocation policy can build long waiting times, even when capacity is sufficient to meet demand. By bringing waiting times down and going away from prioritisation, the waiting list size and average waiting times at the studied department could be maintained almost 90% below the current level – without requiring permanent change in the capacity/demand ratio.

## Background

At the end of 2015, 229 000 patients (4.4% of the population of about 5 million) were waiting for care in secondary health services in Norway.^1^ The mean waiting time for care in somatic specialist health services was about 70 days, while some patients waited for more than a year.^1^ In 2018, the numbers were similar, with 211 788 patients waiting and the mean waiting time for somatic care being 60 days.^2^ Patients waiting for healthcare report more symptoms, poorer health, and reduced health-related quality of life compared to others,^3,4^ and for some, the period of waiting is experienced as devastating.^5^ Some will experience deterioration of health while waiting,^6^ and some have reduced function and ability to work. For some conditions, outcome of treatment seems poorer among patients that have waited longer for care,^7^ and researchers argue that waiting time should be taken into account when considering cost-effectiveness of care.^8^ Shorter waiting times could thus save patients from suffering and health loss, and society from loss of production and costs related to sickness benefits.

However, not all waiting is bad. Waiting lists allow for planning and structuring, both for patients and institutions, and reduce costs related to excess capacity.^9^ Some patients will experience recovery while waiting, thus preventing unnecessary care. However, the administrative costs of keeping waiting lists increase with the size of the list.^10^ Accordingly, for every institution, a certain waiting list size represents the ideal waiting situation. This ideal waiting list is defined as the smallest possible list ensuring maximum capacity utilisation, and as such balancing the needs of the patients (short waiting) and the needs of the healthcare provider (productivity). The average waiting time in this situation defines the ideal waiting time. Many institutions do not know the size of their ideal waiting list, and it seems that the ideal waiting list often is believed to be larger than what is actually the case. Reaching the ideal level could give substantial savings for the institution, and at the same time improve service levels for patients.

Long waiting times for care are often considered an indication of too low capacity.^3,11,12^ However, countries applying resources to increase production and reduce waiting times, often report no lasting effect,^13^ and research has failed to correlate waiting times with hospital resources only.^14^ In Norway, the annual number of new referrals to secondary healthcare has been relatively stable over the last years, and the number of patients waiting does not seem to be increasing.^1^ If resources were insufficient and referrals stable, we would expect an ever-increasing waiting list. The current (2015), stable situation thus indicates capacity sufficient to meet demand.

Factors other than capacity, such as patient logistics, complex booking processes, and poor use of resources, might also influence waiting.^15-20^ There is reason to believe that the policies used for patient appointment allocation in secondary healthcare influence waiting times more than previously assumed.

The simplest way of allocation appointments for start of elective care, is giving each patient the first available appointment. This policy is commonly labelled “first come, first served.” Patient waiting time under this policy is dependent on resource availability only.

However, when waiting time for care is long, “first come, first served” cannot ensure timely treatment for patients requiring care soon. Prioritisation between patients becomes necessary – some patients must receive rapid access, at the expense of others.

There are multiple ways of ordering waiting lists based on priority. Commonly, each patient is evaluated and assigned a priority based on certain criteria. Patients of low priority are given appointments into the future, to make room for higher priority patients arriving later. Patients of highest priority are given no delay beyond that related to resource availability, and are as such granted the first available appointment. Under such prioritisation, waiting time for care is dependent on 2 factors; resource availability and priority.

The procedures used to order waiting lists based on priority are multifold. In recent years, much research has gone into developing and improving procedures and tools for such management (example papers from recent years^21-27^). All such methods, no matter how sophisticated, have in common that criteria-based priorities help define delays in access to care. The benefit for individual patients of high priority is evident – they receive care quickly even when waiting times overall are long.

In Norway, principles for assigning patients priority were established already in 1987.^28^ These principles were later revised in 1997 and 2014,^29,30^ and are now legally reflected in the Patients’ Rights Act.^31^ Every patient referral to secondary healthcare is evaluated by a physician. If the patient is granted access, he or she is given priority to care according to (*i* ) disease severity (prognosis as affected by life expectancy and quality of life), (*ii* ) expected effect of available healthcare, and (*iii* ) the cost-effectiveness of the services. All patients given access to care are also assigned a due date within which care has to be initiated.^31,32^ Routines have been developed to comply with these regulations. A common practice is that the set due date is regarded as the patient’s level of priority, and an appointment is given before, but close to, the due date.

### Aims and Hypotheses

This study had 2 main aims. Firstly, we aimed to investigate whether prioritisation of elective patients for secondary healthcare can be part of the reason for long waiting times. The term prioritisation is here used for any policy where patients are assigned treatment delays based on medical and/or other criteria. We hypothesised that:

- Prioritisation leads to extra waiting.
- Prioritisation can lead to poor utilisation of extra capacity.
- Prioritisation can lead to short-lived effect of extra capacity.

Secondly, we aimed to describe measurements to assess if – and if so, how much – patients have to wait extra at a given department due to prioritisation; and to suggest a process that can be used to reduce unnecessary waiting caused by prioritisation. We hypothesised that:

- The waiting list shape (defined as the number of appointments booked each day from today and onwards) reveals the degree of prioritisation in use.
- The size and shape of the ideal waiting list define a target for waiting list management.
- Changing policy only (without changing the capacity/demand ratio) has no overall effect on a large waiting list.
- When waiting times are long, prioritisations between patients can be necessary in order to achieve timely treatment for patients requiring treatment fast.

The hypotheses were investigated using 3 methods: a simple manual simulation on artificial data, steady-state calculations and dynamic computer simulations on actual waiting list and patient flow data from the Department of Otorhinolaryngology at Haukeland University Hospital, Bergen, Norway in 2015.

For each method we investigated how appointment allocation policies that assign appointments based on priority (PRI) perform compared to not prioritising (giving each patient the first available appointment, NOPRI) with regards to (*i* ) size of waiting list, (*ii* ) cumulative patient waiting time, (*iii* ) cumulative unused capacity, and (*iv* ) shape of waiting list.

## Methods

### Data Material and Operationalization of the Process

For this study, data from the Department of Otorhinolaryngology, Head and Neck Surgery, Haukeland University Hospital, Bergen, Norway were used. This department is the second largest of its sort in Norway, and in 2015 had 27.4 full-time physicians, 60.0 nurses, and in total 143.5 full-time positions. The study covers all new patient cases registered and/or handled during 2015 and their waiting times. During 2015, a total of 13 978 patients were referred to the department. Waiting related to follow-up contacts must be handled differently and was not taken into account.

At the studied department, patients are allocated appointments based on priority. Every new referral is evaluated by a physician. If the patient is given a legal right to care, a due date for start of care is set, based on priority. Finally, scheduling takes place, and the appointment is put down in the appointment diary. Between registration and start of care, patients are part of the waiting list.

To model the above patient flow, 3 variables from the historical data are associated with each patient case: registration date (referral is registered), due date (latest date to start care, based on priority) and start date (care is actually started). The latter represents the end of the patient flow covered in our study.

The variables *waiting time* and *waiting limit* are measured in days and calculated from the above variables. *Waiting time* represents the actual waiting time between registration and start of care, while *waiting limit* represents the upper limit for how long a given patient should wait. This simplified patient and data flow are described in more detail in Supplementary file 1.

### Description of Data Set and Aggregation

The data used consists of 2 parts; the initial waiting list at the beginning of 2015, and the patient flow during 2015. The initial waiting list contains all patients with a *registration date* before January 1, 2015 and either no *start date* or a *start date* on January 1, 2015 or later. The flow data contains all patients with *registration date* , *start date* , or both, during 2015.

The process described above represent a discrete view of the patient flow, where each patient case is followed from one process step to the next. Both the steady-state calculations and the computer simulations performed in Dynaplan Smia^33^ are based on continuous models where discrete patient cases are aggregated into flow rates measured as patients per day.

**Figure 1:**
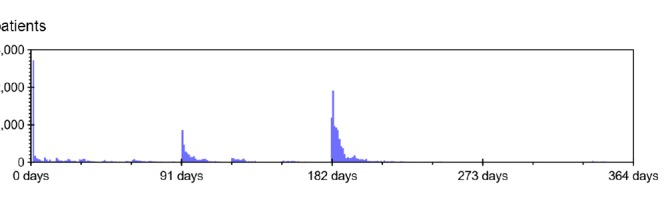
Actual Distribution of Waiting Limits (Maximum Set Waiting Times).

The time dimension used for analyses consists of 365 time intervals of length one day, covering 2015. The waiting limit (maximum set waiting time) is the basis for a second dimension, used for aggregating data into *priority groups:* 0 days, 1 to 90 days, 91 to 181 days, and 182 days and more. These groups represent cases requiring care with different degree of haste. The chosen group boundaries were based on the distribution of waiting limits in the actual data, shown in Figure 1.

Patients who qualified for receiving a due date, but received no such, were placed in the last priority group. Patients in the first group, are acute cases requiring care within 24 hours.

**Figure 2:**
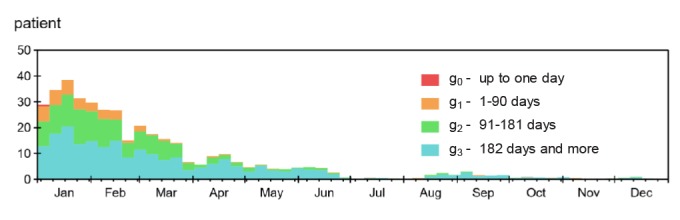
Shape of the Initial Waiting List as Represented by the Appointment Diary. The graph shows the number of patients already scheduled for care in 2015 at the beginning of the year, grouped by week (X-axis) and priority (colour codes). The drop in admissions in February coincides with the “winter vacation” (week 8 of 2015), the drop in March/April coincides with Easter. The period of low activity between mid-June and mid-August coincides with summer vacation.

The department’s appointment diary holds scheduled appointments for today, tomorrow, etc, and can as such be seen graphically as the *shape* of the waiting list. Figure 2 is a snapshot of the *shape* of the appointment diary January 1, 2015. This initial appointment diary holds patients in the initial waiting list, who have a *start date* in 2015, booked before 2015.

Table shows the average waiting time within each priority group and the distribution of patients over the 4 priority groups in our data.

**Table:** Average Waiting Times and Distribution of Patients by Priority Group

**Group**	**Waiting Limit**	**Waiting Time (${\bar{W}}_{g}$)**	**Distribution (D_g_)**
g0	1 days	1 days	16.04%
g1	91 days	30 days	17.79%
g2	182 days	90 days	22.82%
g3	365 days	113 days	43.35%

### Key Model Variables

Formal definitions of the variables are given in Supplementary file 2.

#### Input Variables

Most variables in our models are defined in terms of one or both of the dimensions, *priority group* and *time* , defined above. Subscripts g and t are used to index these dimensions in equations.

*Registration (R):* This variable holds the number of patient cases that are registered for each group and time interval.

*Start care (S):* This variable holds the number of patients who start care for each group and time interval.

*Capacity (C):* The start of care flow depends on 2 factors: number of patients in the waiting list and capacity available to start care. As no information on potential capacity for initiating care per day in 2015 could be obtained, we defined available capacity a given day (t) as the capacity used (S), summed over the priority groups (g).

With this definition of capacity, the actual start care rate (S) in our data acts as an upper limit for the production (healthcare delivery) in the simulation models. The only exception is when simulating the effect of added capacity.

#### Outcome Variables

*Waiting list – size (L) and shape:* The variable *waiting list* holds the number of registered patients waiting for start of care a given day. Depending on analysis, the waiting list size was initialized either from the recorded data at the beginning of 2015, or empty. During the study period, the variable was increased by the flow *registration* (R) and decreased by the flow *start care* (S).

The waiting list size variable has 2 dimensions, *group* and *time* , and measurement unit *patient* .

The *shape* of the waiting list is defined as the number of appointments booked per priority group each day in the interval between today and the end of the appointment diary. This shape is displayed graphically to give a qualitative impression of how the appointment diary is filled.

*Cumulative waiting time (∑W): Cumulative waiting time* holds the number of patient days spent waiting for start of care. The variable is initiated to zero and is increased each day with the size of the waiting list times one day. The variable has 2 dimensions, *group* and *time* , and measurement unit patient*day.

*Cumulative unused capacity (∑U):* The *cumulative unused capacity* is calculated as the cumulative difference between *capacity* rate (C) and *start* rate (S) multiplied by the time step.

### Description of the Simulation Model and the 2 Allocation Policies

Simplified model representations of the real world can be used in retrospective as well as prospective analyses of policies. Through computer-based dynamic simulations, we wanted to learn how waiting and capacity utilisation might have developed at the studied department in response to different policies, different initial conditions, and extra capacity. Departments who want to improve their waiting list situation, can consider using simulation as part of their decision-making process to study likely short, medium, and long-term effects of alternative approaches.

The simulations were performed using Dynaplan Smia,^33^ a stock-and-flow based simulation software. The simulations were performed for 2015 with a time step of 1 day. Recorded daily capacity, registration, start care, and waiting list size and shape were imported into the simulation.

The main stock of the simulation model, the waiting list, holds the sum of all appointments registered in the contained appointment diary variable at all times.

The appointment diary is a 2-dimensional table, where each cell holds the number of patients from a given priority group who are signed up for healthcare a given day. The appointment diary is initialized from the imported data.

The simulation model implements scheduling with and without prioritisation as follows:

*Priority-based scheduling (PRI):* A physician evaluates each patent case and assigns a priority group based on certain criteria. Subsequently, a secretary finalizes the queuing by allocating an appointment time where resources are available and the waiting time is within the waiting limit for the patient’s priority group.

*Scheduling without prioritisation (NOPRI):* A secretary performs the queuing by giving the patient the first available appointment. The scheduling is independent of medical evaluation and no physician needs to be involved.

*Simulation process:* When all patients are given the first available appointment (NOPRI), the simulator searches the appointment diary for the first day where the sum of current appointments is below the capacity that day.

When patients are given appointments based on priority, the simulator starts its search at a distance into the future determined by the average waiting time in the recorded data for the given patient’s priority group (${\bar{W}}_{g}$).

Under both policies the acute cases (g0) are treated within the first day. Our implementation uses re-booking to ensure this. Other approaches can also be used, eg, reserving capacity (which is also supported by the simulation model) or by treating acute cases in separate departments.^34^

Formal definition of the scheduling policies, as well as detailed description of the simulation of appointment allocation is included in supplementary file 3.

## Results

### Manual Simulation on Artificial Data

A simple hand simulation was used to highlight the main characteristics of NOPRI and PRI, and the differences between the waiting lists resulting from these management policies.

**Figure 3:**
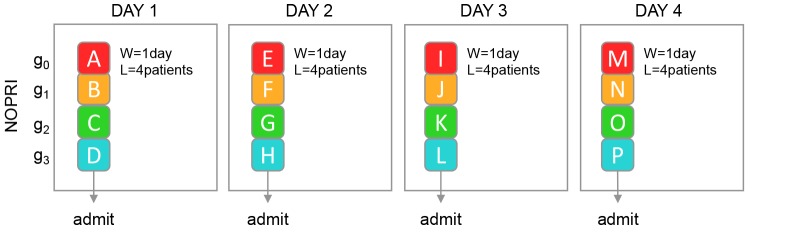
Illustration of NOPRI. The letters A, B, C, etc are used to identify the individual patients. Colours represent priority groups: Red: g0, orange: g1, green: g2, blue: g3. If each patient is given the first available appointment, the entire inflow is handled in one day.

Let us assume that patients are, based on their condition or other characteristics, grouped into 4 priority groups, g0-3, and that exactly one patient from each group arrives each day. We start with an empty waiting list and capacity for start of care equal to demand, ie, 4 patients per day. If each patient is given the first available appointment, all patients are treated the first day (average waiting time ($\bar{W}$) is 1 day) and the waiting list (L) holds 4 patients, as illustrated in Figure 3.

**Figure 4:**
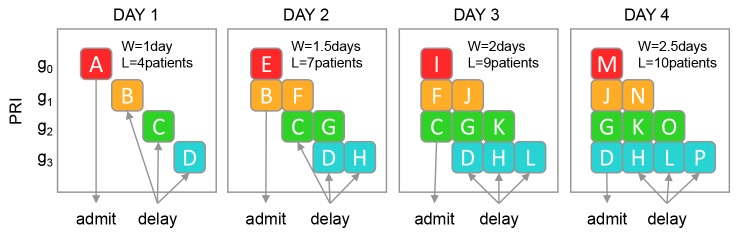
Illustration of PRI. Patients are given appointments into the future, based on priority. The delay for group number *g*_i_ is here set to *i* days. The first day, 4 patients arrive. One (A) gets admitted, while B, C and D get appointments in the future. In day 2, 2 patients (E and B) receive treatment, because they both have reached their due date. The same pattern is followed for day 3 and 4. From day 4 and on the system is in equilibrium.

In Figure 4, we again start with an empty waiting list and 4 patients arriving each day, but patients are now given appointments into the future based on their priority.

When the PRI policy reaches its equilibrium, the waiting list size (L) is 10 patients and the average waiting time (${\bar{W}}_{g}$) 2.5 days.

The illustration also shows that the shape of the waiting list under NOPRI looks like a bar throughout the simulation, while PRI gradually approaches a staircase shape (day 4).

Once steady-state is reached, the same mix of patients are treated every day under both policies, ie, one patient from each priority group receives treatment each day. When prioritisation is not used, all patients get treated as soon as possible; on the first day. When prioritising, one patient from each priority group is still treated each day but patients are systematically treated as late as possible, ie, at their waiting limit.

### Static Calculations on Actual Patient Data

We here determine the policy-specific *minimum* waiting list, the average waiting time and the waiting list shape with the patient flow in perfect equilibrium.

In equilibrium the patient flow is, by definition, the same every day, with no variation. Also, the size of the waiting list (L) is stable, so that the number of treated patients (S) is equal to the number of new referrals (R) each day.

The registration rate in the historical data was 38.3 patients/day, and the start rate was 38.47, indicating a near-equilibrium process. Supplementary file 4 shows further details of a near equilibrium development of the waiting list on an annual basis from 2010-2015, making steady state analyses relevant.

#### Minimum Size of Waiting List

The minimum waiting list is the shortest waiting list ensuring full capacity utilisation in steady-state conditions – thus, when fluctuations in supply and demand are not taken into account (see Supplementary file 2).

Assuming that for elective cases, *registration* must take place at least the day before *start care* , the minimum size of the waiting lists for NOPRI and PRI can be calculated like this:

$$\begin{array}{l} L_{NOPRI}^{min}=R\;day=38.3\frac{patients}{day}=38.3\;patients\\ L_{PRI}^{min}=R\;\sum_{g}{\bar{W}}_{g}D_{g=}2\;872\;patients \end{array}$$

The expression ${\bar{W}}_{g}$ denotes the average waiting time of priority group *g* and *D*_g_ the distribution of each priority group (g). Actual values are available in Table.

#### Minimum Waiting Time

The distribution (D) of patients in the different groups is defined like this:

$$D_{g,t}=\frac{R_{g,t}}{\sum R_{t}}$$

In steady state, registration rate (R), start rate (S), and capacity (C) will be equal, and remain constant. Hence, also the distribution (D) will be constant. Based on the average registration rate (R), the average waiting time () and distribution (*D*_g_) of each priority group (g), the minimum average waiting times for NOPRI and PRI can be calculated like this:

$$\begin{array}{l} {\bar{W}}_{NOPRI}^{\min}=1day\\ {\bar{W}}_{PRI}^{\min}=\sum_{g}{\bar{W}}_{g}D_{g}=75\;days \end{array}$$

Thus, in equilibrium, the smallest possible waiting list is 38.3 patients under NOPRI and 2872 patients under PRI, and the shortest average waiting time is 1 day for NOPRI and 75 days for PRI.

#### Shape of Minimum Waiting List

**Figure 5:**
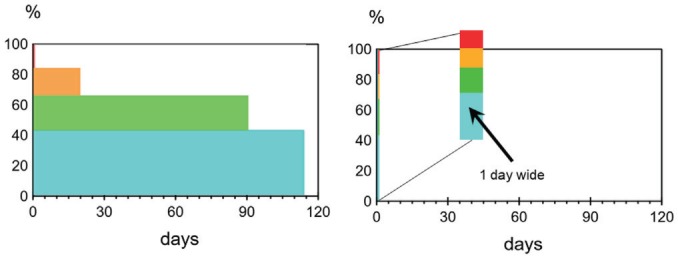
Shape of Steady State Waiting List. Left: PRI. Right: NOPRI.
The graphs show the fraction of capacity that is booked each day between the current date and the end of the appointment diary. Colours represent priority groups: Red: g0, orange: g1, green: g2, blue: g3. The leftmost graph, representing prioritisation, shows the acute cases, g0, as a hardly visible, narrow red bar, stretching from 84% to 100% close to the Y-axis. In the rightmost graph, representing “first come, first served,” all bars are stacked up just next to the Y-axis, showing that all priority groups are handled within the first day.

Under PRI in steady state, a person in group g gets assigned an appointment ${\bar{W}}_{g}$ days after the registration date. The distribution of patients over priority group in our data, gives the waiting list shape to the left in Figure 5. Under NOPRI, all patients receive an appointment the day after their registration, creating the rectangular waiting list shape displayed to the right in Figure 5.

### Dynamic Simulation on Actual Patient Data

#### Starting From an Empty Waiting List

We here compare the dynamic behaviour of the size of the waiting list and the cumulative waiting and unused capacity during the transition from an empty waiting list to the policy-specific ideal waiting list, and subsequently the outcome variables after the initial transition phase.

To determine the ideal waiting list for each policy, and at the same time rule out that large waiting list comes from lack of capacity in the past, we initialized the simulation with an empty waiting list, simulating a department just opening up. In contrast to the minimum waiting list calculated above, which is practically unachievable, as fluctuations in supply and demand are not taken into account, the ideal waiting list calculated here is the smallest waiting list ensuring full capacity utilisation taking the actual historic fluctuations in supply and demand into account (see Supplementary file 2). The inflow was defined by the daily registrations (R) in the recorded data, and daily capacity (C) by the capacity used in the recorded data.

**Figure 6:**
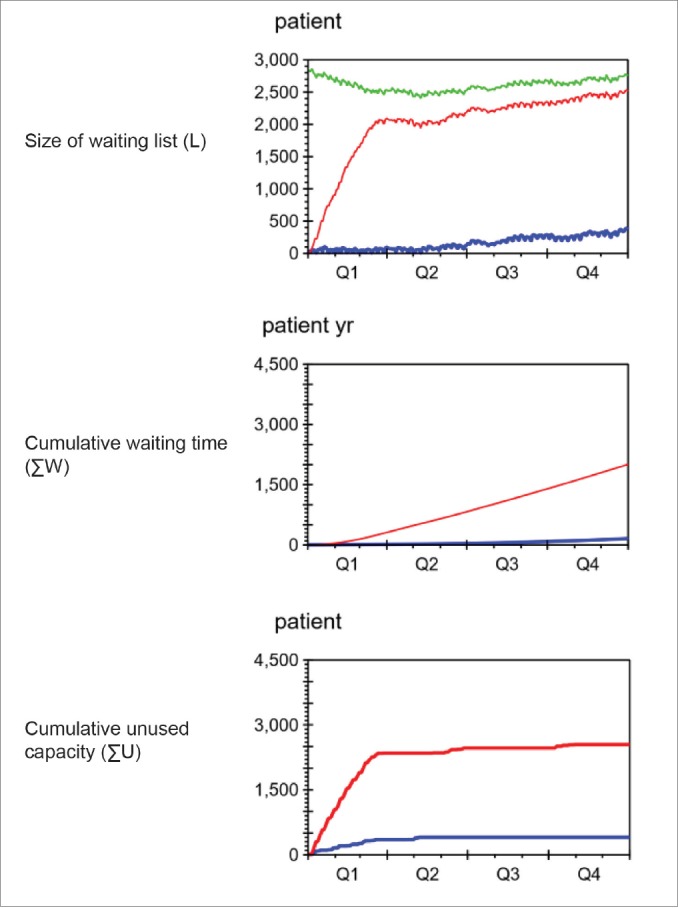
Results of Simulations Started With Empty Waiting List. Red: PRI. Blue: NOPRI. Green: Recorded data.
When prioritising (red line, topmost graph), waiting list size (L) rapidly grew towards the size shown in the recorded data (green line). Without prioritisation the waiting list remained significantly shorter (blue line). The graph in the middle shows significantly more waiting (ΣW) when prioritising (red) than when not (blue). The buttommost graph shows that some capacity was unused (ΣU) during the first quarter (Q1), and that prioritisation (red) led to approximately seven times more loss than without prioritisation (blue).

Figure 6 shows the development of the waiting list size, cumulative waiting time and cumulative unused capacity under PRI and NOPRI.

#### Size of Waiting List

For both policies, capacity was lost during the first part of the simulation - due to lack of patients. This led to an increase in waiting list size until the policy-specific ideal size was reached. We see that the ideal waiting list sizes are not completely constant but follow fluctuations in the net patient flow.

The recorded waiting list (green) for 2015 started with 2824 patients and ended with 2760 patients. The decrease of 64 patients (2.26%) over the year shows that the department spent enough capacity (C) on initiating care (S) to balance the inflow of new patients (R) during the year, and reduce the waiting list somewhat.

L _recorded _= 2760 patients

Under NOPRI (blue) the waiting list (L) grew slowly towards a stable level of approximately 370 patients, far below the recorded data (green) and the level when prioritisation was used (red).

$$L{\;}_{NOPRI}^{ideal}=371\;patients$$

Under PRI (red) the waiting list grew quickly during the first 3 months, and slowly approached a level close to the recorded waiting list size at the end of 2015.

$$L{\;}_{PRI}^{ideal}=2515\;patients$$

#### Average Waiting Time

The second graph in Figure 6 compares the development of cumulative waiting time with and without prioritisation. Under NOPRI, very slow growth in cumulative waiting time was seen. In contrast, PRI led to accelerating growth for the first 3 months, and a quite steep linear growth from then on. At the end of the simulation, PRI had resulted in 2008 patient years of waiting, while NOPRI had given 156 patient years of waiting (92% lower than PRI).

In a near steady state, average waiting time for patients can be approximated like this:

$$\bar{W}=L/S$$

Since both policies reach an approximate steady state towards the end of the period, the ending size of the waiting list together with the average capacity (S = C = 37.46 patients per day) can be used to compute the average waiting time without and with prioritisation:

$$\begin{array}{l} {\bar{W}}_{NOPRI}^{ideal}=\frac{L_{NOPRI}^{ideal}}{S}=\frac{371.80pas}{\frac{37.46pas}{day}}=10days\\ {\bar{W}}_{PRI}^{ideal}=\frac{L_{PRI}^{ideal}}{S}=\frac{2515.70pas}{\frac{37.46pas}{day}}=67days \end{array}$$

#### Unused Capacity

The buttommost graph in Figure 6 compares the development of cumulative unused capacity under PRI and NOPRI. When starting from an empty waiting list, 401 patients less than the capacity for the year were treated under NORPI, and 2545 patients less were treated under PRI. This gives a capacity utilisation of 97% under NOPRI and 82% under PRI. Both policies ran out of patients in the beginning, leading to capacity loss, until the waiting list reached the policy-specific ideal level. PRI required a much larger waiting list than NOPRI before fully utilising capacity.

#### Shape of Ideal Waiting List

**Figure 7:**
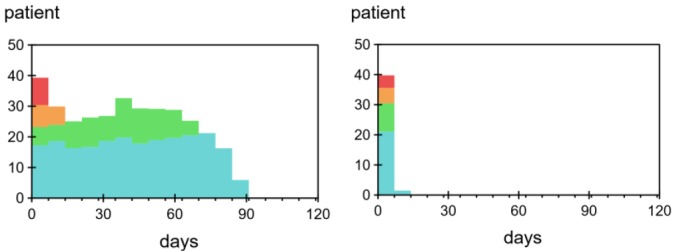
Shapes of Waiting List After Initial Phase. Left: PRI. Right: NOPRI.
The graphs show the fraction of capacity that was scheduled for each day between the current date and the end of the appointment diary. Colours represent priority groups: Red: g0, orange: g1, green: g2, blue: g3. The leftmost graph, representing prioritisation, shows that appointments were booked further and further into the future for each priority group. In the rightmost graph, representing “first come, first served,” all bars were stacked up next to the Y-axis, showing that most patients were handled within the first week, and the rest within the second.

When the cumulative unused capacity levelled off and no further capacity loss was seen, each policy had reached its ideal level. The shapes of the ideal waiting lists under PRI and NOPRI are shown in Figure 7.

The shape is much wider under PRI than NOPRI. The colouring shows that patients of the highest priority have short waiting times under both policies, while patients of lower priority experience shorter waiting times under NOPRI.

In our model, weekly averages were used to avoid clutter from periodic drops in capacity on weekends. We also simplified the allocation of appointments by selecting a time around the average for each priority group. This led to a narrower appointment diary than the one seen in the recorded data – where appointments were spread significantly more out, in a wide staircase-shape extending for a full year (and beyond) (Figure 2 above).

### Starting From the Initial Recorded Waiting List

We here compare the waiting list, waiting time, and capacity utilisation of the 2 policies when starting the simulations from the waiting list recorded January 1, 2015. With this initialization, the waiting list started out higher than the ideal waiting list size for both policies. Hence, no capacity loss due to lack of patients was expected. Apart from the waiting list initiation, the set-up for this analysis was equal to that of the analysis above.

**Figure 8:**
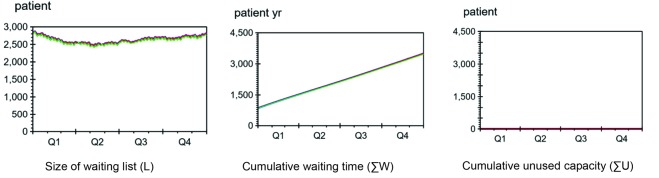
Results of Simulations Starting With Recorded Waiting List. January 1, 2015. Red: PRI. Blue: NOPRI. Green: Recorded data.
Each graph shows coloured lines on top of each other, thus, change in waiting list management policy had no effect on waiting list size (L), amount of waiting (∑W) and capacity loss (∑U) when starting the simulation from recorded situation at beginning of 2015.

Figure 8 shows the development of the size of the waiting list, the cumulative waiting time and the cumulative unused capacity when starting with the recorded waiting list.

#### Size of Waiting List

With regards to waiting list size, the simulations performed very similarly to the actual data for 2015, whether prioritising or not. At the end of 2015, the waiting lists sizes for NORPI, PRI, and recorded data were:

L _NOPRI _= 2796 patients

L _PRI_ = 2796 patients

L _recorded_ = 2766 patients

#### Average Waiting Time

Also cumulative waiting time was nearly identical to that seen in the historic data. The average waiting times for PRI and NOPRI were found to be 75 days, the same as in the recorded data.

$$\begin{array}{l} {\bar{W}}_{NOPRI}=\frac{L_{NOPRI}}{S}=\frac{2796.60pas}{\frac{37.46pas}{day}}=75days\\ {\bar{W}}_{PRI}=\frac{L_{PRI}}{S}=\frac{2796.60pas}{\frac{37.46pas}{day}}=75days\\ {\bar{W}}_{recorded}=75days \end{array}$$

In this situation, the average waiting time is larger than the lower waiting limit of the most urgent non-acute priority group (g1), which according to Table covers the interval from 1 to 91 days. Abandoning PRI in this situation would lead to breaches of waiting time limits for patients in this group. Acute cases (g0), would not be affected, because patients in this group are always treated on the day of arrival.

#### Capacity Utilisation

Capacity was fully utilised under both PRI and NOPRI.

### With Increased Capacity

**Figure 9:**
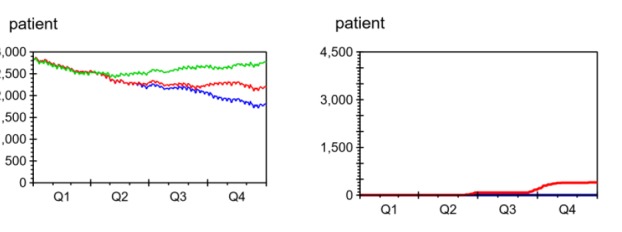
Effect of Adding Resources. Red: PRI. Blue: NOPRI. Green: Recorded data.
The graph to the left shows that for both policies the waiting list (L) was reduced compared to the actual data (green). Prioritisation (red) led to roughly half the reduction seen when not prioritising (blue). The graph to the right shows that some capacity was unused (ΣU) when prioritising, while capacity was fully utilised when not (blue).

We here compare the waiting list size and capacity loss of the 2 policies when capacity was increased above demand. The simulation setup was identical to the simulation starting with the actual waiting list, except that the capacity was set 10% higher for the 3 last quarters of the simulation period (amounting to a total of 1006 extra patients) (see Figure 9).

#### Effect of Added Capacity

With the added capacity, the waiting list under NORPI dropped from 2796 patients to 1798 patients, a reduction of 1006 patients. This represents full utilisation of added capacity. Under PRI, the number of patients on the waiting list was reduced from 2796 patients to 2188, a reduction of 608 patients. The recorded waiting list ended at 2760 patients (no capacity was added here; this is historical, recorded data).

#### Utilisation of Added Capacity

While no capacity was lost under NOPRI, PRI utilised only 60% of the added capacity.

## Discussion

### Summary of Findings

Our study shows that prioritisation can indeed be part of the reason why many patients experience long waiting times for healthcare. The term prioritisation is here used for any policy assigning elective patients treatment delays for secondary healthcare based on medical and/or other criteria. With this definition, the manual simulation, the static calculations and the computer-based simulations on actual patient data all showed that prioritisation, compared to giving each patient the first available appointment, leads to extra waiting. Indeed, the waiting when prioritising was 7 times longer than when not. The simulations also showed that prioritising can lead to poor resource utilisation and short-lived effects of extra capacity.

The study further shows that the shape of the appointment diary can reveal whether a department is prioritising patients for care. The appointment diary is bar-shaped when not prioritising, while a wider, stair-case like shape reveals prioritisation. The difference between the current waiting list at the department and the ideal waiting list when not prioritising, can further show what can be gained by abandoning prioritisation. However, simply changing policy when waiting times are long cannot bring average waiting times down. Such a change could also lead to breaches of waiting limits; prioritisation between patients might be necessary when waiting times are long.

### Prioritisation Builds Waiting

All 3 methods used in our study showed that prioritisation leads to extra waiting. Indeed, static calculations gave waiting times 75 times longer when prioritising than when not.

When simulating a department just opening up, with no patient back-log, prioritising built waiting times similar to those seen in Norway today (2015-2018). Within a year, the average waiting times when prioritising reached 67 days. The corresponding recorded average waiting time at the department was 75 days and the national average in 2015 was 70 days (all somatic care).^1^ Comparatively, when not prioritising, the waiting time at the end of the year averaged at 10 days. As these simulations were started from an empty waiting list, with capacity equal to demand, the resulting wait cannot be caused by present or previous lack of capacity.

All policies for appointment allocations have an inherent, ideal level, where patient flow fluctuations and composition, and policy-specific characteristics no longer result in capacity loss. When the waiting list is below this ideal level, resources are only partly utilised (as shown in our simulations). Thus, if the waiting list is brought below the ideal level by a temporary increase in capacity, the effects will be temporal only. Once capacity again equals demand, the waiting list will grow towards, and stabilize at, this level. Our simulations show that the ideal waiting list was 7 times larger when prioritising than when not. This means that, compared to a department that prioritises, a department with the same patient flow that does not prioritise, could keep the waiting list 85% smaller – using the same resources.

Our simulations also showed that when capacity was increased above demand, prioritisation utilised only 60% of the extra resources. These findings can together explain why achieving significant and lasting improvement in the waiting situation when starting with long waiting lists can be difficult. In 2013, the Organisation for Economic Co-operation and Development (OECD) published a review of policies used by 13 countries to bring waiting times down.^13^ Only one of the countries (The Netherlands) reported a successful transition into a situation where waiting times were no longer considered an issue. Countries applying more resources to increase production in general reported no lasting effect.^13^ Poor return on investment, and improvement being temporary only, can be explained if the countries studied were prioritising patients for care.

### Can Our Findings Be Generalised?

Our results are in large based on data from a single, Norwegian hospital department, applying a specific priority-based appointment allocation policy. Can they be generalized to other departments using appointment allocation policies postponing care for some patients to leave room for future patients with higher priority?

The manual simulation highlights how *any* policy postponing care for some patients will generate waiting – inherently. When supply and demand are in balance, this can also be proven mathematically (see Supplementary file 5 for details). For prioritisation to give a waiting list as short as the one when not prioritising, the average waiting time for each priority group must be exactly one day. Consequently, the algorithm for prioritising can only have a single priority group with 1 day as its waiting limit. The algorithm has degenerated into non-prioritisation; no patients are postponed, and each patient is given the first available appointment. The essence of this reasoning is that the minimum size of the waiting list will always be longer when prioritising than when not. As these findings are inherent to any policy delaying care for certain patients, our results are generalizable, qualitatively, to any department using such policies.

Studying different prioritisation policies at different departments will not give results completely equal to ours. The ideal waiting list size inherent to any priority-based policy depends on many factors, for instance patient fluctuations and delay chosen for each priority group. Priority policies can also be made more advanced to reduce capacity loss. For instance, one could work to avoid capacity loss by looking for holes in the appointment diary and filling these by calling patients who can come in at short notice. As mentioned in the introduction, much research goes into developing and improving procedures and tools for priority-based scheduling.^21-27^ Such efforts can bring capacity loss towards zero, and as such towards – but never past – the capacity utilisation when giving each patient the first available appointment.

In addition to the drawbacks described above, prioritisation consumes administrative and medical resources for evaluation of condition and appointment allocation. Deciding on appropriate priority level or waiting time can be challenging,^21,35,36^ and in spite of prioritisation, care is not always initiated within the due date,^1^ not even for cancer patients.^11^ Pushing appointments into the future creates longer waiting times and longer waiting lists, which must be administered. The administrative costs of a waiting list increase with the size of the list,^10^ and larger lists increase the likelihood of patients requiring reevaluation. Thus, the keeping of unnecessary long waiting lists diverts efforts away from patient care, further decreasing capacity.

Further, in its extreme form, prioritisation treats all patients as late as (legally or medically) possible. The manual simulation shows that both policies treat the same mix of patients every day. Hence, and maybe contrary to expectations, the delay of low-priority patients does not lead to treatment of more high-priority patients.

Also, when maximum waiting limits are introduced, waiting times below the limits might be understood as “good performance.” If the number of waiting limit breaches is used as a key performance indicator, or even as the basis for incentives or sanctions, waiting may start drifting towards the maximum limit. This seems to be the case in our region, where patients’ actual waiting times often are very close to the assigned maximum.^37^ This indicates a need for new performance indicators.

With such drawbacks, one can wonder why prioritisation is ever used. When waiting times grow past the waiting limit for patients with haste, prioritisation of patients becomes necessary to enable timely treatment and avoid waiting limit breaches. The major advantage of prioritisation, and the very reason it is used, is that patients requiring treatment fast will get it in time, even when waiting times overall are long.

### Reducing Unnecessary Waiting by Changing Policy

Not all departments with long waiting times are prioritising. Factors other than poor appointment allocation policies can of course also contribute to long waiting times. Such factors are discussed in Supplementary file 6.

Departments with long waiting times for start of care, can perform a simple test to see if the problem might be linked to prioritisation. If a snapshot of the department’s current waiting list resembles the characteristic staircase shape, this indicates prioritisation.

If the department is prioritising, the gain to be expected if prioritisation is successfully set aside can be estimated by comparing the current waiting list size at the department to the ideal size when not prioritising.

However, the specific order of patients in a queue does not influence the length of the queue. When waiting lists are larger than the ideal waiting list for both policies, abandoning prioritisation without bringing capacity above demand cannot reduce waiting list size. Also, if waiting times are long, simply giving each patient the first available appointment would not ensure timely care for urgent cases.

In such situations, prioritisation must be set aside, *and* the waiting list brought down by temporarily bringing capacity above demand, until all waiting times are below the lowest, relevant waiting limit. During the transitional phase, care must be taken to ensure timely treatment of patients requiring treatment fast.

When the new equilibrium has been reached, all patients wait approximately the same time for care. The waiting time will remain short for high priority patients, while low priority patients will experience significantly shorter delays. Both policies treat acute cases first.

When the average waiting time is short, most – if not all – patients will receive care within their set waiting limit, and no prioritisation is necessary. Due to fluctuations and stochastic variations in supply and demand, situations might arise where a patient’s due date is before the first available appointment. Such cases must be handled differently, for instance by reserving dedicated capacity, by re-booking other patients, or by treatment in separate departments.^34^

Our study indicates that the size and shape of the waiting list in relation to its ideal size and shape, can act as performance indicators in assessing quality of elective healthcare delivery at the departmental, hospital, regional, and national level. The ideal size and shape can act as targets for waiting list management at all levels.

A more detailed description of a process that can be used to reduce unnecessary waiting by changing policy, is included in Supplementary file 7.

### Strengths and Limitations

#### Three Complementary Analyses

Three different approaches – a manual simulation, steady state calculations, and dynamic simulations on actual patient data – were used to compare waiting list size, waiting time, and capacity utilisation when prioritising and not prioritising. The results complement each other, strengthening our conclusions. We also found that the simulation results were not very sensitive to choice of intervals for the priority groups (results not shown).

Though dynamic simulations can be used to show likely effects of policy changes, they can, of course, not predict the future.

Our results give clear indications of the benefits of changing away from prioritisation, but the process suggested for the transition has yet to be tested out. Currently, some departments at Haukeland University Hospital, Bergen, Norway are planning to move away from prioritisation, following the procedures presented in this manuscript and in Supplementary file 7.

#### The Ideal Waiting Time When not Prioritising Might Be Too Short

As mentioned in the introduction, waiting time for care must be sufficiently long to allow for planning of resources and communication with patients. Such considerations must be made at the individual department in order to determine how close to the ideal size and shape it is possible to come with current technology, patient mix, etc.

#### Our Study Concerns Acute Cases and Elective Patients, Follow-up Is not Covered

Policies for allocating appointments for start of care and follow-up must differ. First appointment is often wanted as soon as possible. Follow-up appointments, on the other hand, must come after a certain time interval. More research is needed in order to evaluate waiting times for further appointments, and how management of both first and follow-up appointments can be optimized.

#### Waiting From Referral to Start of Care Is Considered

Patients wait for care at multiple stages within the healthcare sector, both at the community and hospital level. Once referred to secondary healthcare, there is waiting to be taken in for the first time, then there might be waiting for medical imaging, surgery or other clinical investigation or treatment. The total time waited until healthcare can be considered fulfilled (if ever) is thus a combination of multiple waits, and is not studied here.

#### Quality of Data and Routines

Generally, the quality of register data of waiting times is considered uncertain, and in particular, there are differences in practices for setting the end point of waiting time. The end date for waiting can for instance be when the specialist starts investigation of the patient (for unclear cases) or when treatment is started (for fully diagnosed patients).^38^ In practice, the end of waiting time is typically registered at first hospital contact.^38^

#### Potential Capacity not Obtainable

The use of recorded production during 2015 as a proxy for potential, available capacity prevents our simulation model from performing better than the recorded data. However, this limitation does not change the conclusions of our study. In fact, if capacity is increased above demand, our simulations show that not prioritising would outperform prioritising even more.

#### Many Variations of Prioritisation

Waiting lists and waiting times are in this study evaluated based on data from one department only. Planning profiles^37^and waiting times^1^ seem to differ between specialties, and as mentioned above, much research goes into developing and improving prioritisation based policies (example papers^21-27^).

Our implementation of prioritisation is an example of a general queuing policy where patients with low priority have to wait longer than patients with high priority. By changing the parameters of our prioritisation model, it is possible to reduce any gap between recorded data and simulation results for any department implementing a form of such prioritisation. We did not aim to exactly match our models to the data of a particular department, but to show that any policy delaying certain patients to make room for future patients will inherently generate waiting.

#### Barriers to Change

Some physicians and researchers worry that efforts for reducing waiting times are doomed to fail, as demand will increase when healthcare becomes more accessible. At the institutional level, reduced demand for healthcare can lead to more patients being taken in for controls or more frequent follow-ups.^39^ When waits become shorter, patients who previously would not have met the criteria for secondary healthcare, might be referred. Research on elasticity is conflicting. For instance, data from the United Kingdom show low elasticity of demand with respect to waiting time, suggesting that increased resources may reduce waiting times without greatly stimulating demand.^40^ In Australia, however, demand for care has been found to be highly responsive to waiting time.^41^

It has also been claimed that the major barriers against introducing “first come, first served-based” appointment allocation policies in the primary care setting “are the fear of change and the lack of confidence that existing resources can meet the demand for care.”^18^ Knowing and applying best evidence are 2 very different dimensions in the complex world of healthcare systems. Effectively creating change is both time and resource consuming, and requires thoughtful approaches.^42-44^

## Conclusion

When waiting time for care is long, giving each patient the first available appointment cannot ensure timely care for patients requiring treatment fast. Prioritisation between patients becomes necessary. However, prioritising elective patients for start of care can build and maintain long waiting times – even when capacity is equal to demand. Further, poor return on investment and temporary effect only are often seen when capacity is increased. Lasting improvement may require a change in the way appointments are allocated.

If waiting lists are brought sufficiently down, giving each patient the first available appointment can ensure timely care for all elective patients. At the studied department, not prioritising maintained waiting lists and average waiting times almost 90% below today’s level – without requiring resources beyond what is used for care today.

Focus on maximum waiting times – introduced as guidelines to improve the waiting situation – might be driving waiting times up, towards the set maximum. Our study indicates that the size and shape of the waiting list in relation to its ideal size and shape, can act as performance indicators in assessing quality of elective healthcare delivery. Further, the ideal size and shape can act as targets for waiting list management.

Although prioritisation can be done in many ways, it can never perform better with regards to average waiting times than simply granting each new patient the first available appointment. Attention should be shifted away from optimising prioritisation, towards how “first come, first served” can be used for scheduling initial appointments for elective patients in secondary healthcare.

## Acknowledgements

The authors want to thank Pål Ove Vadset, head of Section for Health Service Development at Helse Bergen, for initiating and leading the project where Dynaplan was engaged to help improve the waiting list situation at hospitals in the Helse Vest region.

We also want to thank all the other members of the project team for their insight and contribution. In particular, we are in debt to the 2 advisors from the above-mentioned section, Audun Lange and Håkon Ersland, who prepared the data from the Patient Flow Database (Norwegian: “Forløpsdatabasen”) in a format that could be used in our study.

We are grateful to Finn Olav Sveen, Solution Specialist at Dynaplan, who has helped with validation and quality assurance of the simulation models used in our study.

Many thanks to Malcolm Doupe, Associate Professor at University of Manitoba, Winnipeg, MB, Canada for valuable feedback on the manuscript.

## Availability of data and material

The data used in this study are collected in an Excel workbook, available for download at http://www.myrtveit.com/research-data/haukeland-head-and-neck-2015.xlsx.

## Ethical issues

This study is part of a larger, ongoing project – the patient flow database (Norwegian: “Forløpsdatabasen”) – at Haukeland University Hospital, Bergen, Norway aiming to monitor and improve treatment and patient flow. The Patient Flow Database was approved by the Data Protection Officer (ref: 2012/6943), as was the present study.

## Competing interests

The authors declare that they have no competing interests. The basis for this study is a simulation model developed by Dynaplan, where Myrtveit is executive partner, as part of a consulting engagement for Helse Vest and Helse Bergen, funded by Helse Vest, Helse Bergen, and Dynaplan together. The research and writing of the article was done after the project was finished, without external funding.

## Authors’ contributions

MM had the idea for the study and is responsible for the study design. SMMS, TH and JHH contributed in further refining the study design. MM conducted the analyses. All authors took part in the interpretation and discussion of results. SMMS and MM wrote the first draft. All authors contributed in further shaping the manuscript. The final version was approved by all authors.

## Authors’ affiliations

^2^Department of Health Promotion, Norwegian Institute of Public Health, Bergen, Norway. ^2^Department of Clinical Science, University of Bergen, Bergen, Norway. ^3^Department of Research and Development, Haukeland University Hospital, Bergen, Norway. ^4^Department of Clinical Medicine, University of Bergen, Bergen, Norway. ^5^Clinic of Surgery, Haukeland University Hospital, Bergen, Norway. ^6^Dynaplan AS, Manger, Norway (https://www.dynaplan.com/en/).

## Supplementary Files


Supplementary file 1. The simplified patient and data flow.

Supplementary file 2. Formal variable definitions.

Supplementary file 3. Formal definitions of scheduling policies and details of the simulation process.

Supplementary file 4. Long-term development of waiting list.

Supplementary file 5. Mathematical proof of that prioritisation leads to extra waiting.

Supplementary file 6. Other factors that might influence waiting.

Supplementary file 7. A process for reducing unnecessary waiting.

## Key messages

**Implications for policy makers**

- To reduce the risk of entering a vicious circle of increasing waiting times, policies assigning certain patients delays for care, in order to make room for patients of higher priority arriving later, should not be introduced.
- To make the transition from long waiting times towards shorter, capacity must be brought above demand until patients can be served in order of arrival.
- To get lasting effect of initiatives to reduce patients’ waiting time, the use of prioritisation must be abandoned (or reduced to a minimum). Our results indicate that the concept of ideal size and shape of the waiting list could be useful as the basis for target setting and incentives for waiting list management in secondary healthcare.

**Implications for the public**

Findings from this study can be applied to achieve significant reduction in waiting times for new patients who are referred from primary to secondary healthcare.

Long waiting times can have many causes, including lack of resources. Once waiting times have been allowed to grow above a certain limit, prioritisation between patients becomes necessary to avoid breaches of maximum waiting times. This study shows that policies assigning low-priority patients delays for care, in order to make room for patients of higher priority arriving later, build long waiting times. At the studied department, simply giving each patient the first appointment available, could keep waiting times at 10 days instead of 75, without requiring permanent increase in resource use.
