# Supplementary material for: Long Waiting Times for Elective Hospital Care – Breaking the Vicious Circle by Abandoning Prioritisation
Source: Int J Health Policy Manag. 2019 Oct 30;9(3):96–107. doi: 10.15171/ijhpm.2019.84 (PMC7093047; doi:10.15171/ijhpm.2019.84)
Supplement: Supplementary file 1 — The simplified patient and data flow. [file ijhpm-9-96-s001.pdf]

Supplementary file 1. The simplified patient and data flow

The studied patient and data flow start when a patient referral is registered at the hospital and ends when care starts.

After registration of a new referral, a physician evaluates the case and sets a due date for care, based on priority. Subsequently, scheduling takes place. Between *registration* and *start care*, patients are part of the *waiting list*.

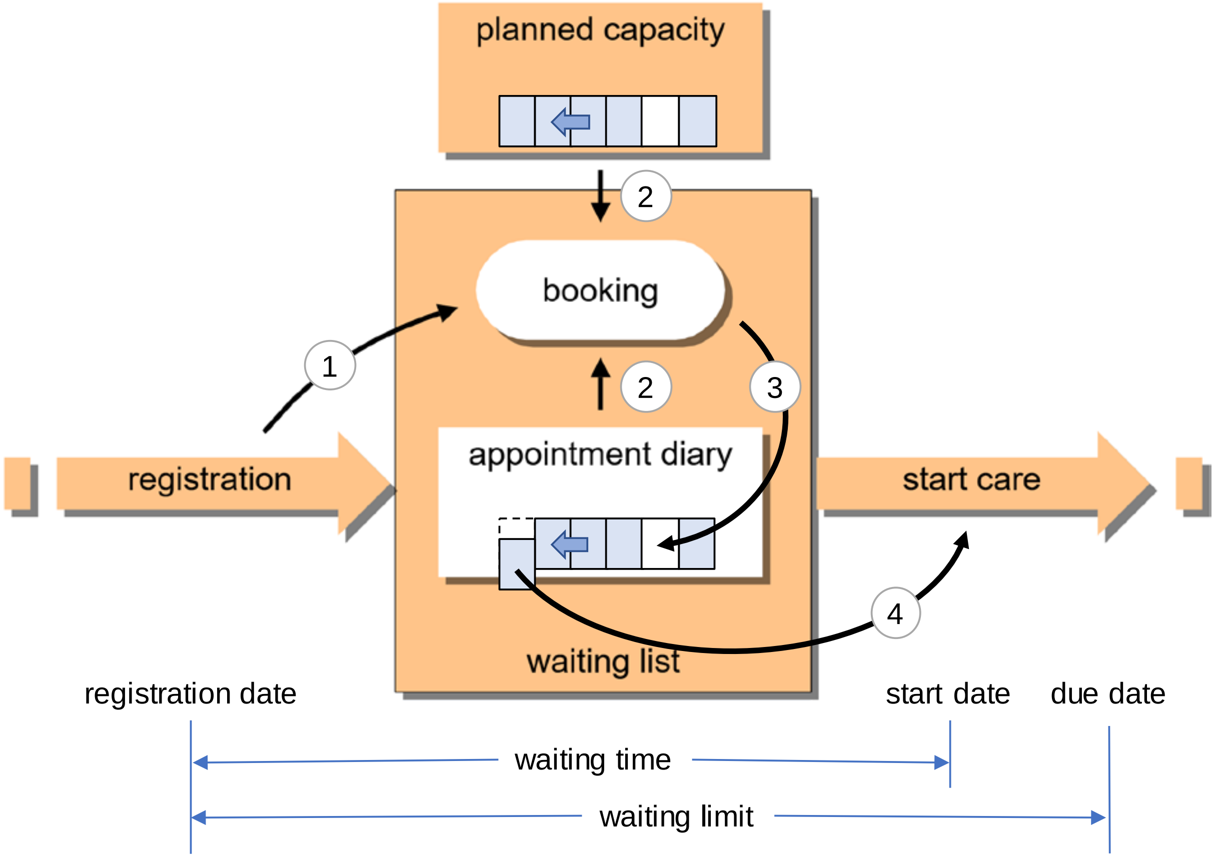

Figure 1 - Patient and data flow

The *continuous* version of the patient flow is represented by the inflow *registration*, the stock *waiting list*, and the outflow *start care*. The orange arrows represent average daily flow rates, measured as *patients/day*.

The *discrete* version of the patient flow is represented by the numbered arrows. Starting from the point of *registration* (1), every individual patient is followed throughout the process. The *planned capacity* and the *appointment diary* are checked (2), and a *booking* is made for a future date where

capacity is available (3). *Start care* (4) is signalled when the booked appointment takes place. At this time the patient leaves the *appointment diary* and the *waiting list?*

The *appointment diary* holds scheduled appointments for today, tomorrow, etc, represented by the boxes inside the *appointment diary* in the figure. The arrow (3) from *booking* to *appointment diary* shows an example where a patient is given an appointment 4 days into the future.

At the end of every day, *planned capacity* and *appointment diary* are shifted one day to the left, as illustrated by the 2 left-pointing arrows (⇦).

As shown in the bottom of the figure, *waiting time* and *waiting limit* are calculated from the historic data associated with each case; registration date (referral is registered), due date (latest date to start care, based on priority) and start date (care is actually started).
