# Supplementary material for: Long Waiting Times for Elective Hospital Care – Breaking the Vicious Circle by Abandoning Prioritisation
Source: Int J Health Policy Manag. 2019 Oct 30;9(3):96–107. doi: 10.15171/ijhpm.2019.84 (PMC7093047; doi:10.15171/ijhpm.2019.84)
Supplement: Supplementary file 2 — Formal variable definitions. [file ijhpm-9-96-s002.pdf]

Supplementary file 2. Formal variable definitions

Most variables in our models are defined in terms of one or both of the dimensions, *priority group* and *time*, defined in the manuscript. Subscripts g and t are used to index these dimensions in equations.

Δt represents the time step of the time dimension. It has the value 1 day in our models.

Input variables

Registrations,

$$R_{g,t} = \text{sum of cases with waiting limit} \in \text{g and registration time} \in \text{t.}$$

Start of care,

$$S_{g,t} = \text{sum of cases with waiting limit} \in \text{g and start time} \in \text{t.}$$

Capacity, sum of start care over all groups at time t:

$$C_t = \sum_g S_{g,t}$$

Initial waiting list,

$$L_{g,t0} = \text{initial number of patients registered but not yet served.}$$

Outcome variables

Waiting list size; initial list size plus integral of net flows over time:

$$L_{g,T} = L_{g,t0} + \sum_{t=t0}^{T} \left(R_{g,t} - S_{g,t}\right) \Delta t$$

Cumulative waiting; integral of waiting list size over time:

$$\Sigma W_{g,T} = \sum_{t=t0}^{T} L_{g,t} \Delta t$$

Cumulative unused capacity; integral of capacity minus production over time:

$$\sum U_T = \sum_{t=t0}^{T} \left(C_t - \sum_{g} S_{g,t} \right) \Delta t$$

Average waiting time in steady state (where L, R and S are constant):

$$\bar{W}_g = L_g / S_g$$

Distribution of patients; percentage of patients in each priority group:

$$D_g = \frac{\sum R_g}{\sum R}$$

Shortest waiting list that fully utilizes capacity

Static model without prioritisation:

$$L_{NOPRI}^{min} = R\ day$$

Static model with prioritisation:

$$L_{PRI}^{min} = R \sum_{g} \overline{W}_g\ D_g$$

Dynamic model including fluctuation, without prioritisation:

$$L_{NOPRI}^{ideal} = determed\ by\ simulation\ starting\ from\ empty\ waiting\ list$$

Dynamic model including fluctuation, with prioritisation:

$$L_{PRI}^{ideal} = determed\ by\ simulation\ starting\ from\ empty\ waiting\ list$$
