# Supplementary material for: Long Waiting Times for Elective Hospital Care – Breaking the Vicious Circle by Abandoning Prioritisation
Source: Int J Health Policy Manag. 2019 Oct 30;9(3):96–107. doi: 10.15171/ijhpm.2019.84 (PMC7093047; doi:10.15171/ijhpm.2019.84)
Supplement: Supplementary file 3 — Formal definitions of scheduling policies and details of the simulation process. [file ijhpm-9-96-s003.pdf]

Supplementary file 3. Formal definitions of scheduling policies and details of the simulation process

Priority-based scheduling (PRI)

A given patient is assigned an appointment a distance $W = q(A, p(Cr))$ into the future. The letters W, q, A, p, and Cr are defined as:

W – Waiting time
q – Queuing function
A – Resource availability
p – Prioritisation function
Cr – Criteria

This represents a practice where a physician carries out the prioritisation process (p) based on the criteria (Cr) and assigns the patient a priority group (g). A secretary finalizes the queuing (q) by booking an appointment at a time where resources are available (A) and the waiting time (W) is within the waiting limit for the patient's priority group.

Priority groups are ordered in descending priority, starting with g0; the acute cases. We matched our models to the data by using the recorded, average waiting time, $\overline{W}_g$, for each group as the target waiting time for that group.

Scheduling without prioritisation (NOPRI)

Any given patient is assigned an appointment a distance (W) into the future, defined as:

$W = q(A)$; where
W – Waiting time
q – Queuing function
A – Resource availability

As the scheduling is independent of medical evaluation, no physician needs to be involved. A secretary performs the queuing (q) by giving the patient the first available appointment.

Simulation process

The simulations follow the patient flow process described below.

1. For each new patient case *registration* (R), determine *priority group* (g) from the waiting limit.
2. Sort new patients by priority group in descending order.
3. For every new patient, book an appointment like this:
   - 3.1. Determine the *available capacity* (A) = *planned capacity* - *appointment diary*.
   - 3.2. Determine the patient's waiting time $W=q(A,g)$, where q is the chosen queuing policy (with or without prioritisation).
   - 3.3. Increase *appointment diary* (g,W) by one patient.
