# Supplementary material for: Long Waiting Times for Elective Hospital Care – Breaking the Vicious Circle by Abandoning Prioritisation
Source: Int J Health Policy Manag. 2019 Oct 30;9(3):96–107. doi: 10.15171/ijhpm.2019.84 (PMC7093047; doi:10.15171/ijhpm.2019.84)
Supplement: Supplementary file 4 — Long-term development of waiting list. [file ijhpm-9-96-s004.pdf]

Supplementary file 4. Long-term development of waiting list

Recorded data for the years 2010 through 2015 for the studied department show that the inflow (R) and outflow (S) of patients are nearly in equilibrium, with a fluctuation of ±4% change in the net annual flow.

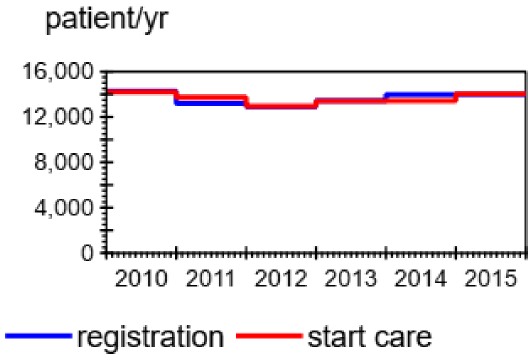

t (year)	S – R (patients)	(S-R)/R (%)
2010	-71	-0.5 %
2011	542	4.1 %
2012	44	0.3 %
2013	-28	-0.2 %
2014	-552	-4.0 %
2015	64	0.5 %
Sum	1	

Figure 1 - Long-term developments in inflow and outflow

The changes in net flow are below 1% for all years, except 2, where the changes in net flows almost cancel each other out (2011 and 2014: 4.1% - 4.0% = 0.1%).

As shown in Figure 2, opening and closing values for the six-year period are only off by one patient, and the net flow (S-R)/R for the studied period (2010-15) is just 0.5%. Based on these observations, a steady-state analysis seems quite applicable for the chosen department and year.

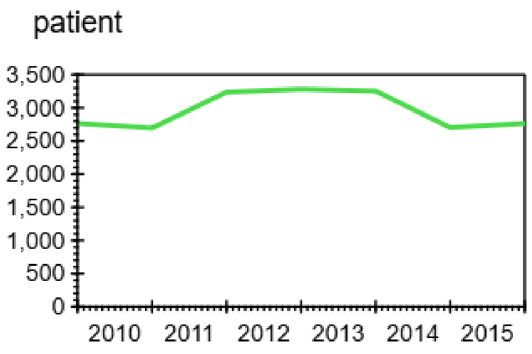

t (date)	L (patients)	$(L_t - L_{t0})/L_{t0}$ (%)
01.01.2010	2767	0 %
01.01.2011	2696	-3 %
01.01.2012	3238	17 %
01.01.2013	3282	19 %
01.01.2014	3254	18 %
01.01.2015	2702	-2 %
01.01.2016	2766	0 %

Figure 2 - Long-term development of the waiting list
