# Supplementary material for: Long Waiting Times for Elective Hospital Care – Breaking the Vicious Circle by Abandoning Prioritisation
Source: Int J Health Policy Manag. 2019 Oct 30;9(3):96–107. doi: 10.15171/ijhpm.2019.84 (PMC7093047; doi:10.15171/ijhpm.2019.84)
Supplement: Supplementary file 5 — Mathematical proof of that prioritisation leads to extra waiting. [file ijhpm-9-96-s005.pdf]

Supplementary file 5. Mathematical proof that prioritisation leads to extra waiting.

Let us start by looking at the size of the waiting list (L) in steady state. In Supplementary file 2 we have defined the shortest possible waiting list under prioritisation like this:

$$L_{PRI}^{min} = R \sum_{g} \overline{W}_g \, D_g$$

Under the assumption that there is at least one day between registration (R) and the start of care, and capacity is equal to demand, the minimum waiting list size without prioritisation is:

$$L_{NOPRI}^{min} = R \; day$$

As reasoned in the main manuscript, for prioritisation to give a waiting list as short as the one when not prioritising, the average waiting time for each priority group must be exactly one day. Consequently, the algorithm for prioritising can only have a single priority group with 1 day as its waiting limit. The prioritisation part of the algorithm is therefore no longer in effect; the policy has degenerated into “first come, first served”.
