# Supplementary material for: Long Waiting Times for Elective Hospital Care – Breaking the Vicious Circle by Abandoning Prioritisation
Source: Int J Health Policy Manag. 2019 Oct 30;9(3):96–107. doi: 10.15171/ijhpm.2019.84 (PMC7093047; doi:10.15171/ijhpm.2019.84)
Supplement: Supplementary file 6 — Other factors that might influence waiting. [file ijhpm-9-96-s006.pdf]

Supplementary file 6. Other factors that might influence waiting

In the following we discuss factors other than appointment allocation policies that often are proposed to cause waiting.

Waiting due to insufficient capacity

As mentioned in the introduction, insufficient capacity is often given as an explanation for long waiting times.[1,2,3] Insufficient capacity would over time lead to an ever-increasing waiting list. The data we have studied show that resources for care and referrals have been in good balance over recent years (see Supplementary file 4). Following this, the long waiting times observed cannot be explained by a current lack of resources. Still, lack of capacity might be highly relevant for waiting times in other situations, times and places.

Waiting due to lack of capacity in the past

If long waiting times are caused by lack of capacity, but capacity has been equal to demand during the last years, we must have previously gone through a period with demand exceeding capacity. A temporary growth in the waiting list due to such an imbalance would leave the waiting list large indefinitely, unless brought back down by an imbalance in the opposite direction. Today, with capacity equal to demand, we have enough capacity to treat all referred patients, but not enough to bring the waiting lists down.

If the lack of capacity in the past has caused today's long waits, the problem could be fixed by temporary increasing capacity over demand. Subsequently, once the waiting list had been reduced, all referred patients could be treated, like today, and using the same resources, but practically without waiting time (given no increase in demand). Our results deem this strategy unlikely to work in Norway today. Firstly, prioritisation gives poor return on investment. Secondly, when capacity is brought back to equal demand, any improvement seen in waiting list size (beyond the ideal level) will be lost as the list grows back to its inherent – and high – ideal level.

Waiting in order to reduce inflow

Long waiting times reduces availability and attractiveness of healthcare services. In a market perspective, this can lead to reduced demand. In cases where treatment is not necessary or would have no (justifiable) effect, it might be tempting for the hospital to give patients appointments far into the future. When knowledge about long waiting times spreads, referrals of patients with such conditions might decline. Long waiting times become a means to an end; namely to reduce the inflow.

However, if a patient is entitled to healthcare for his or her condition, there is no reason for the patient to wait unnecessarily. Using long waiting as a means for reducing the inflow would in such cases be unethical.

Waiting in expectance of recovery

Some patients recover without healthcare. Time spent in expectance of spontaneous recovery is strictly not correctly labelled as waiting time for care. When waiting times are short, the GP can delay referring cases where spontaneous recovery is likely until it becomes clear whether secondary healthcare is needed. When expected waiting time for healthcare is long, however, the GP might, in order to save the patient from excessive waiting if the condition does not improve, refer the patient to secondary healthcare immediately. As such, and counter intuitively, long waiting times can lead to more referrals.

Waiting as a result of measuring

When maximum waiting limits are introduced, waiting times below the limits indicate "good performance". If the number of breaches of a waiting limit becomes a key performance indicator, or even the basis for incentives or sanctions, waiting may start drifting towards the maximum limit. This seems to be the case in our region, where patients' actual waiting times often are very close to the assigned maximum.[4]

Furthermore, if hospitals are evaluated by the fraction of patients treated in time, one might worry that patients whose waiting limit is already passed are not prioritised. Data from the UK have shown a drop in the probability of being admitted to hospital once the patient has passed the government set maximum waiting time.[5] There is no reason to believe that the patients' conditions suddenly improved once the waiting limit was passed. However, once the target is missed, the extent to which it is breached might be less important, and resources might be directed elsewhere. Data from Norway might indicate the same tendency: Ten percent of all patients experiencing a breach of allocated waiting time for the somatic specialist health services, wait 60 further days for start of care.[6]

Finally, waiting times might also be kept unnecessary long if hospitals are awarded funding on the basis of long waiting lists.[7]

References

1. Siciliani L., Borowitz M., Moran V., *Waiting Time Policies in the Health Sector: What Works?* 2013: OECD Publishing.
2. Larsen, B., *Ventelister, helsetap og sykehuskapasitet.* Tidskrift for Den norske legeforening, 2001. **19-20**(121): p. 22555.

3. Brustugun, O.T., Nome, O., Bruland, Ø. S., Ottestad, L., Lilleby, W., *Brystkreftpasienters vurdering av ventetid før strålebehandling*. Tidskrift for Den norske legeforening, 2003. **123**: p. 1685-1686.
4. Gangstøe, J., Heggestad, T., Norheim, OF. , *Norwegian Priority Setting in Practice – an Analysis of Waiting Time Patterns Across Medical Disciplines.* Int J Health Policy Manag, 2016. **5**(6): p. 373.
5. Dimakou, S., et al., *Identifying the impact of government targets on waiting times in the NHS*. Health care management science, 2009. **12**(1): p. 1-10.
6. Fugleset, A., Karstensen, A, Håndlykken, EK, *Ventetider og pasientrettigheter 2015*, in *IS-2448*. 2016, Helsedirektoratet.
7. Iversen, T., *A theory of hospital waiting lists.* Journal of Health Economics, 1993. **12**(1): p. 55-71.
