# Supplementary material for: Long Waiting Times for Elective Hospital Care – Breaking the Vicious Circle by Abandoning Prioritisation
Source: Int J Health Policy Manag. 2019 Oct 30;9(3):96–107. doi: 10.15171/ijhpm.2019.84 (PMC7093047; doi:10.15171/ijhpm.2019.84)
Supplement: Supplementary file 7 — A process for reducing unnecessary waiting. [file ijhpm-9-96-s007.pdf]

Supplementary file 7. A process for reducing unnecessary waiting

In this section we suggest a process that departments can use to reduce any unnecessary waiting caused by prioritisation, and that healthcare managers can use to measure and steer towards set targets.

G.1 – Process improvement

Some public hospital departments in the Helse Vest region of Norway are currently (in 2018/19) considering testing out the findings from this study to see if this can lead to reduced waiting times for patients, better use of resources, and less stress on employees. In relation to this, a process for making the transition away from prioritisation was developed. A summary is presented below.

Phase 1 – Analyse the as-is situation

Any department where patients experience long waiting times for start of care, can perform simple tests to see if the problem might be linked to the use of prioritisation. Real data, of the nature we have used in this study, are the basis for measurements that can be used to assess the current situation.

First, look at the shape of the current appointment diary. If it looks like a bar, prioritisation is not in use, and the reason for long waiting time lies elsewhere.

Then, check if the waiting list size (L) has been stable over the last year or so. If the list is growing or declining, there is an imbalance between supply and demand. Investigate the development in registrations (R), start of care (S), and capacity utilisation (U) to identify why the waiting list is not in steady state. If demand (R) grows over time, a corresponding increase in production (S) will be necessary to avoid a worsening of the problem.

Next, investigate the composition of the patient flow and group patients into priority groups based on waiting limits. If there are no hard waiting limits, there is no need to prioritise.

With the priority groups in place you can produce the patient flow outcome variables across time and group (waiting list size (L) and shape, average waiting time ($\overline{W}$), and capacity utilization (C)). These are defined in the main text and Supplementary file 2. Run a simulation, or use another approach, to compute the ideal waiting time when not prioritising.

The difference between current waiting time at the department and ideal waiting time when not prioritising represents the potential reduction you can achieve by abandoning prioritisation. If

the values are very similar, there is not much to gain, except for reduced cost and effort of your booking process.

Phase 2 – Prepare the transition

Involve all stakeholders (including patient representatives) who will be involved in, and influenced by, the change.

To address the risk of waiting limit breaches, compare the ideal waiting time to the smallest waiting limit (other than for acute cases). If there are limits below the ideal waiting time, permanent measures must be put in place to serve this group. As an example, dedicated capacity can be reserved.

The transition away from prioritisation involves a period where supply must exceed demand. Together with the stakeholders, identify options. Can resources be increased, productivity improved, workload shifted, demand reduced? Would new or better use of equipment and rooms help? Use of simulation – or other tools – can help quantify the effect of initiatives alone and in combination, and to forecast the expected time needed to complete the transition.

This phase should lead up to a plan for the transition and how different stakeholders will contribute and be affected. New routines and new technology, or new use of current technology, should be ready before entering the next phase.

Phase 3 – Implementation and monitoring

Put the initiatives and routines defined in the preparation phase into action. Measure performance (like in phase 1) on a monthly basis and compare to plan. Address gaps and make adjustments to plan and/or implementation.

Phase 4 – The new normal

The transition is done. Any extra resources provided for the transition can be withdrawn. Monitor the actual situation compared to the ideal on a regular basis, to ensure that waiting times do not start drifting upwards – a situation that can easily lead to waiting time breaches and re-introduction of prioritisation – basically, taking us back to start.

G.2 – Reflections over targets and incentives

There are many stakeholders involved in and influenced by the quality of waiting list management. Patients (and society) benefit the most from short waiting times, and should as such, maybe be more involved in decisions that influence waiting time. In our meetings with hospitals about potential reductions in waiting list size, the most enthusiastic by far, have been

the secretaries dealing with bookings. They tell about daily stress relating to difficult trade-offs, lack of vacant appointments, painful communication with patients, etc.

For stakeholders with more decision power, existing incentives for change might not be sufficient, and might even work in the wrong direction. As an example, long waiting lists can be an argument for increased funding. Our study, together with other research, shows that long waiting lists can be a result of poor waiting list management. When this is the case, increased resources might not solve any problems – unless policies are also changed.

This study suggests measurements departments can use to compare the shape and size of their waiting lists to the ideal. These measurements could potentially also be used by healthcare managers to assess the quality of waiting list management at different levels; national, regional, hospital, department.

One might also consider introducing such measurements to set targets and create incentives/sanctions relating to waiting list management at all levels of the healthcare system. There would be 3 rationales behind switching to such measurements:

1) Incentivising waiting list management of high quality.

2) Avoiding bad side-effects of existing measurements.

3) Bringing focus away from *how* the services are delivered (use of prioritisation) to the *quality* of the services (timely treatment of patients, efficient use of resources, good working conditions for employees). Focusing on the results would also open up for creativity and local adaptations of policies to find the best solutions for reaching the desired quality standard.
